# Avoiding Glaucoma Surgery Through Allergen Identification by Skin Testing: A Case Report

**DOI:** 10.7759/cureus.90969

**Published:** 2025-08-25

**Authors:** Takahiro Suzuki, Naoyuki Serita, Yosuke Miyao, Takashi Matsushima, Muneyuki Inosaka

**Affiliations:** 1 Department of Ophthalmology, Tokai University School of Medicine, Kanagawa, JPN

**Keywords:** glaucoma, goldmann applanation tonometer, intraocular pressure, lidocaine, scratch test

## Abstract

Drug-induced allergic reactions are unpredictable because almost all pharmaceuticals can act as allergens. Accurate identification and avoidance of suspected drugs are critical to prevent serious adverse events. Glaucoma is a chronic condition that causes irreversible vision loss and requires ongoing intraocular pressure (IOP) monitoring and management. This report describes the case of an 83-year-old man with elevated IOP in the left eye due to exfoliative glaucoma. The patient had a history of cataract surgery and allergic reactions to polyvinyl alcohol-iodine eye drops, lidocaine, and oxibuprocaine hydrochloride (Benoxil). IOP was monitored using non-contact tonometry. A dermatologist's skin prick test (also referred to as a scratch test in Japan) showed that lidocaine was the only drug that caused an allergic reaction before the surgical intervention. This allowed for the safe reintroduction of benoxil and the accurate measurement of IOP using Goldmann tonometry. Accurate IOP readings with Goldmann applanation tonometry optimized medical therapy to avoid surgery. Allergy testing can enhance glaucoma management through reliable IOP assessments. This highlights the importance of proactively identifying allergenic drugs for lifelong glaucoma care.

## Introduction

Drug allergies remain unpredictable and can be elicited by a wide range of medications, posing an ongoing challenge in the clinical setting. Allergic reactions, including anaphylaxis, have been reported occasionally in ophthalmic preparations, such as eye drops [1]. Drug allergies associated with the use of Goldmann applanation tonometry (GAT), namely, hypersensitivity to fluorescein dye or topical anesthetics, are considered uncommon in routine practice. In such cases, the use of alternative tonometers that do not require topical agents is recommended. Allergic contact dermatitis from ophthalmic medications is generally rare, with an incidence of approximately 0.7-1% in patch-test registries; however, positivity rates can be higher in specific cohorts, such as up to ~13% among patients using β-blocker eye drops [2]. Immediate hypersensitivity to fluorescein dye has also been reported, though only rarely, in isolated case reports [3]. Thus, although the frequency of such reactions is low, drug allergies that interfere with the performance of GAT remain a clinically relevant concern, necessitating careful evaluation and appropriate management. Therefore, it is crucial to accurately identify the causative agent and avoid its administration to prevent potentially severe outcomes.

Glaucoma is a chronic, progressive optic neuropathy that results in irreversible loss of vision. Preserving visual function requires long-term treatment guided by precise and reliable measurements of intraocular pressure (IOP) [4].

However, in some cases, the use of GAT, the gold standard for assessing intraocular pressure, may be restricted due to medication allergies. In these situations, non-contact tonometry can serve as an alternative; however, its lower accuracy and reproducibility compared to GAT may compromise the reliability of measurements and potentially influence clinical decision-making [5].

This report describes a case of glaucoma in which collaboration with dermatologists enabled the identification of a safe topical agent through a skin prick test. This enabled accurate measurement of intraocular pressure using GAT, allowing for appropriate therapeutic adjustments and, ultimately, the avoidance of surgical intervention.

## Case presentation

Informed consent was obtained from the patient for the publication of this case report and accompanying images as per the requirements of our institution’s ethics policy. An 83-year-old man with elevated intraocular pressure (IOP) in the left eye was referred to our department. The patient had undergone bilateral cataract surgery at another institution in June 2016. In November of the following year (2017), his IOP was 22 mmHg in the right eye and 38 mmHg in the left eye. Treatment was initiated with latanoprost (Xalatan) and brinzolamide (Aizopt) in both eyes. Additional medications, including timolol maleate, ripasudil hydrochloride, and brimonidine tartrate, were subsequently prescribed; however, each of these topical agents induced adverse effects, such as conjunctival hyperemia, pruritus, and blepharitis, leading to their discontinuation. Latanoprost (Xalatan) was the only medication continued. Despite the continued use of latanoprost, IOP remained uncontrolled, with readings of 10 mmHg in the right eye and 39 mmHg in the left eye. The patient was referred to our department in March 2024 for further evaluation and management.

His medical history included interstitial pneumonia, for which he was receiving home oxygen therapy, and allergic reactions following cataract surgery. In both cases, 25 mg of hydroxyzine hydrochloride (Atarax P) was administered orally before cataract surgery. The cataract surgeries proceeded without complication, and no intraoperative allergic reactions were observed. However, approximately six hours postoperatively, the patient developed fever, agitation, and facial flushing, requiring emergency hospitalization. Anaphylaxis was suspected based on the clinical course and response to treatment. Consequently, several agents were considered potential allergens, including latex, lidocaine with adrenaline (xylocaine), povidone-iodine, and oxybuprocaine hydrochloride (benoxil), and their use was contraindicated. Regarding the exclusion of Benoxil in this case, at the previous clinic, intraocular pressure was measured with a non-contact tonometer before cataract surgery, and the patient required emergency hospitalization postoperatively due to a suspected anaphylactic reaction. Therefore, when glaucoma was later suspected, the possibility that Benoxil might represent a potential allergen was taken into consideration, and its use was deliberately avoided.

**Figure 1:**
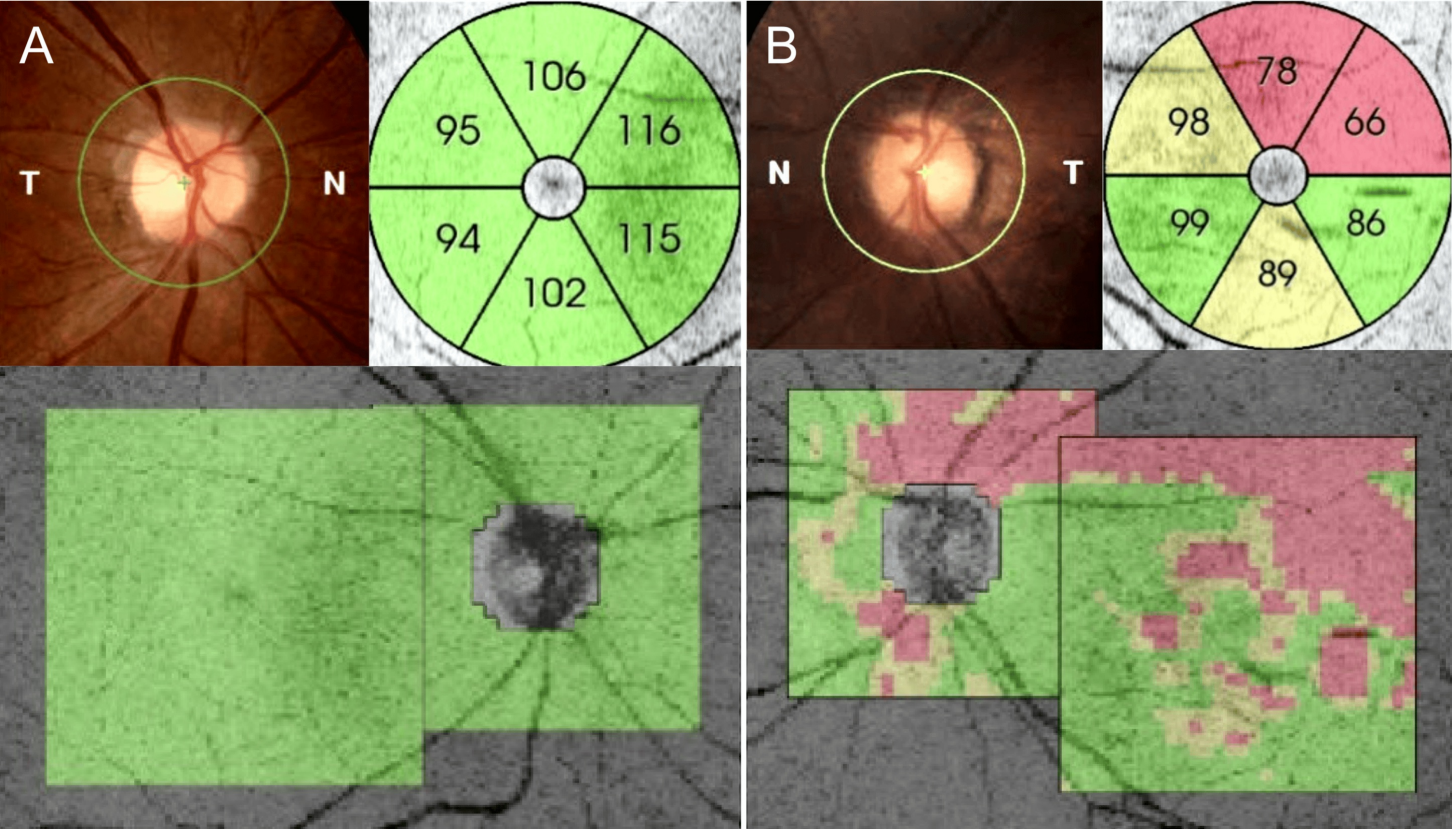
Comparison of optical coherence tomography images of both eyes In the right eye, the retinal nerve fiber layer (RNFL) appears intact around the optic disc and macular area, with thickness measured in micrometers (µm) (A). In the left eye, RNFL thinning was evident in the superotemporal region of the optic disc and superior macula, with thickness measured in micrometers (µm) (B).

**Figure 2:**
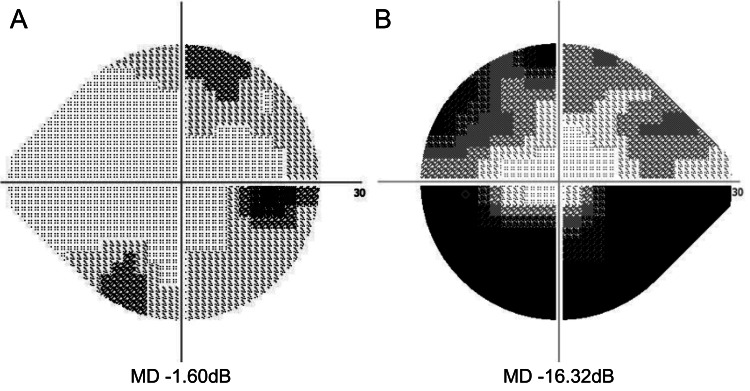
Humphrey field analyzer (HFA) results of both eyes HFA revealing no abnormalities in the right eye (A). In the left eye, defects that corresponded to the areas of retinal nerve fiber layer thinning observed on optical coherence tomography were detected (B).

The patient had no relevant family history. On initial examination at our facility, the best-corrected visual acuity was 1.0 in the right eye and 0.7 in the left. IOP measured using i-Care tonometry was 13 mmHg in the right eye and 34 mmHg in the left eye. The anterior segment findings included signs of exfoliation syndrome in the left eye, and both eyes were pseudophakic with no signs of conjunctival allergy. Fundoscopy revealed a glaucomatous optic disc cupping in the left eye. Optical coherence tomography (OCT) revealed thinning of the retinal nerve fiber layer in the superior region of the left eye, as illustrated in Figure 1. Humphrey visual field testing demonstrated defects corresponding to this area, as shown in Figure 2. For treatment, the patient, who had previously experienced an adverse event during cataract surgery, strongly wished to avoid surgical intervention. Therefore, although aware of its limited effectiveness, the patient continued treatment with once-daily instillation of latanoprost in both eyes.

Acetazolamide (Diamox) 250 mg twice daily was added, which reduced IOP in the left eye to the mid-teenth range. However, due to the potential need for early glaucoma surgery and the associated risks of both local and general anesthesia in this patient, dermatology consultation was requested to identify safe medications. In this case, considering the patient’s advanced age and history of severe allergic reactions, we selected the skin prick test as the initial screening method because it posed a lower risk. A skin prick test was performed, in which minor abrasions were made on the forearm after applying the test solutions, and the results were assessed 15 minutes later. The test revealed a positive reaction to lidocaine, but no adverse reactions to benoxil, povidone-iodine, and saline.

As the skin prick test for Benoxil was negative, Benoxil eye drops were administered four weeks after the patient visited our hospital, followed by a 24-hour observation period. Therefore, Benoxil eye drops were administered, and the patient was monitored over a 24-hour period. No allergic symptoms were observed. This allowed for the monitoring of intraocular pressure using Goldmann applanation tonometry (GAT). The patient was subsequently able to discontinue oral Diamox therapy and was being managed with topical therapy alone, without the need for surgical intervention. The patient had been reluctant to undergo surgery, and thus, this outcome was considered satisfactory from the patient’s perspective.

## Discussion

Allergic reactions to local anesthetics cause anaphylaxis in approximately 1% of cases [6]. In some instances, a biphasic reaction may develop within 6-12 hours following the initial onset of symptoms [7], with the nature and severity of the secondary response varying considerably from the initial presentation [8]. In the present case, the patient developed consistent allergic symptoms, including fever, agitation, and facial flushing, approximately six hours after cataract surgery in 2016, and a skin prick test performed in 2024 confirmed a positive reaction to lidocaine. Although it is difficult to establish a definitive causal relationship, these clinical and laboratory findings support the likelihood that the symptoms observed in 2016 were attributable to lidocaine, which was later identified as an allergenic substance. Moreover, these findings suggest the possibility of an IgE-mediated immediate hypersensitivity reaction, potentially accompanied by a biphasic course. Such secondary reactions may range in severity from mild to more severe than the initial episode [9]. It is also known that antihistamines may suppress cutaneous manifestations such as flushing and urticaria. However, their use may delay the recognition of allergic responses, and delayed treatment is associated with an increased risk of biphasic anaphylaxis [10]. In this case, routine preoperative administration of hydroxyzine (Atarax P) may have masked early symptoms and delayed their manifestation. Nonetheless, the clinical presentation and treatment response were consistent with anaphylaxis. An important point to consider is the potential for reactions attributable to the muscarinic (anticholinergic) effects of Atarax P. However, in the present case, we regard this possibility as unlikely. Hydroxyzine is an H1 receptor antagonist, which generally acts to suppress, rather than provoke, histamine-mediated responses. Moreover, the facial flushing and fever observed in this patient are more plausibly explained by the release of mediators such as histamine, prostaglandins, and cytokines from mast cells, rather than by the adverse effects of hydroxyzine.

From an ophthalmologic standpoint, accurate IOP assessment is essential for the effective long-term management of glaucoma and preservation of visual function. GAT is the most reliable method for measuring the IOP [11]. However, owing to the patient’s allergy history and contraindications to topical anesthetics, GAT could not be performed initially. Therefore, IOP must be monitored using non-contact tonometry, which is known to have limitations in both accuracy and reproducibility.

In this case, collaboration with the dermatology department enabled us to perform a skin prick test, which revealed a positive reaction to lidocaine and a negative response to benoxil. This allowed for the safe reintroduction of Benoxil and resumption of IOP measurements using GAT. No allergic symptoms recurred after the use of Benoxil, and accurate IOP monitoring allowed appropriate therapeutic adjustments. The patient was ultimately able to discontinue systemic medication (acetazolamide) and has since maintained stable IOP control with topical therapy alone.

Severe allergic reactions to local anesthetics and topical ophthalmic medications are rare; however, once they occur, they may lead to serious consequences. Although it is not practical to perform allergy testing for all patients, previous reports have indicated that those with a history of allergic reactions are more likely to develop additional drug allergies [12]. This case demonstrates that allergy to local anesthetics can complicate even routine ophthalmic procedures such as intraocular pressure measurement. Furthermore, it underscores the clinical value of allergy testing and highlights the importance of interdisciplinary collaboration, including cooperation with dermatologists, in expanding treatment options. In patients with similar allergic histories, early dermatological evaluation may contribute to safer and more effective glaucoma management and help maintain quality of life.

## Conclusions

In this case, dermatological allergy testing was not performed for all ophthalmic agents, such as eye drops, viscoelastic materials used during cataract surgery, intraocular lenses, intraocular irrigation fluids, and disinfectants, because standardized test preparations were not available and their reliability and safety had not been established. However, by conducting skin testing in response to the allergic reaction and narrowing down the suspected agents, more accurate intraocular pressure control and treatment were possible. Given that glaucoma requires lifelong management, collaboration with dermatologists can be beneficial in cases where suspected drug allergies limit the therapeutic options.
